# Element accumulation performance of living and dead lichens in a large-scale transplant application

**DOI:** 10.1007/s11356-020-11797-7

**Published:** 2020-12-04

**Authors:** Elva Cecconi, Lorenzo Fortuna, Marco Peplis, Mauro Tretiach

**Affiliations:** 1 Department of Life Sciences, University of Trieste, Via L. Giorgieri 10, 34127 Trieste, Italy; 2 Department of Chemical and Pharmaceutical Sciences, University of Trieste, Via L. Giorgieri 1, 34127 Trieste, Italy

**Keywords:** Lichen transplants, Bioaccumulation, Devitalization, Interpretative scale, *Pseudevernia furfuracea*

## Abstract

In bioaccumulation studies, sample devitalization through acid washing or oven drying is commonly applied to enhance the element accumulation efficiency of moss sample. Such aspect, however, has never been considered in biomonitoring surveys using lichens. In this study, the trace element accumulation performance of living (*L*) and dead (*D*) samples of the lichen *Pseudevernia furfuracea* was compared by a side-by-side transplanting at 40 sites in a large, mixed land use area of NE Italy for 8 weeks. Devitalization was achieved without any physico-chemical treatments, by storing lichen samples in a dark cool room for 18 months. Health status of lichens was assessed before and after the sample exposure by chlorophyll fluorescence emission. Although elemental analysis of the two exposed sample sets revealed a similar trace element pollution scenario, the content of 13 out of the 24 selected elements was higher in *D* samples. By expressing results as exposed-to-unexposed (*EU*) ratio, *D* samples show a higher bioaccumulation signal in 80% of transplant sites for Al, Ca, Fe, Hg, Pb and Ti. Overall, the health status of lichen samples might lead to interpretational discrepancies when *EU* ratio is classified according to the recently proposed bioaccumulation scale.

**Supplementary Information:**

The online version contains supplementary material available at 10.1007/s11356-020-11797-7.

## Introduction

Lichens and mosses are highly performing bioaccumulators, which provide reliable information on the source apportioning of airborne elements and their depositional patterns (Giordano et al. 2013). For this reason, their use is frequently recommended as complementary to conventional monitoring by instrumental devices (Marć et al. 2015).

The wide application of biomonitoring techniques by lichens and mosses over years triggered a major research interest for the processes underlying metal accumulation (e.g. Garty et al. 1979; Brown and Beckett 1985; Tyler 1989; Vázquez et al. 1999). These processes may be very complex, as many factors affect the element accumulation by biological systems (or even by their individual symbionts, in case of mutualistic associations; Bačkor and Loppi 2009). However, in spite of such interest and the growing supportive role of biomonitoring in environmental forensics and decision-making processes, the research aimed at enhancing the methodological consistency of biomonitoring techniques has often followed separated pathways for mosses and lichens. This produced unbalanced outcomes in terms of available protocols, supra-regional sampling networks, data quality and comparability (Cecconi et al. 2019a). A perfect illustration of this phenomenon is represented by the investigation of trace element bioaccumulation in relation to the vitality of the biomonitor. As a matter of fact, such an aspect was frequently addressed in the framework of active “bryomonitoring” (i.e. biomonitoring by moss transplants, of which the moss bag technique is the most used approach, e.g. Aničić et al. 2009a, b; Basile et al. 2009; Giordano et al. 2009; Debén et al. 2016), whereas it has scarcely been faced for lichens.

To date, it is acknowledged that devitalization of moss gametophytes enables an enhanced efficiency of contaminant capture by passive uptake processes (see Ares et al. 2012 and references therein). Especially, the particulate interception and entrapment at the surface level is enhanced in dead mosses (Giordano et al. 2013), with useful effects in terms of achievable trace element pollution signals. Further advantages of devitalizing samples would consist in the reduced variability of results at site level (Gailey and Lloyd 1986; Castello 1996) due to the absence of (i) metabolic activity (Giordano et al. 2009; Capozzi et al. 2017) and (ii) growth during the exposure period (which is a non-negligible source of data variability; Fernández et al. 2009; Fortuna and Tretiach 2018). In this light, the leitmotif of sample devitalization has been carried forward with great consistence in the bosom of bryomonitoring, as reflected by the ‘Mossphere’, a highly standardized exposure device of recent development which uses devitalized shoots of an axenically cultured *Sphagnum palustre* clone (Reski et al. 2016).

Differently, the influence of lichen vitality on the efficiency of elemental accumulation was addressed in a single field work. Indeed, Adamo et al. (2007) assessed the accumulation performance of the macrolichen *Pseudevernia furfuracea* (L.) Zopf in comparison to that of the moss *Hypnum cupressiforme* Hedw. in a 6-week transplant experiment carried out in two Italian sites with different pollutant loads and climatic conditions. Besides performing an inter-species comparison, the authors demonstrated that living *P. furfuracea* samples did not show a better performance with respect to dead ones (Adamo et al. 2007).

Irrespective the test species, in most methodological studies targeting the issue of biomonitor vitality in relation to bioaccumulation, devitalization is generally carried out by acid washings and/or oven-drying (Ares et al. 2012). Acid washing (or “activation”) consists in rinsing the material in an acid medium, with the aim of leaching metal ions from the cell walls, hence regenerating the cation exchange sites to increase the bioconcentration capacity (Brown and Wells 1988; Brown and Brown 1991; Adamo et al. 2007). This procedure notably deteriorates the tissues (Giordano et al. 2009). In oven-drying, the material is simply maintained at temperatures higher than 100 °C for 24 h; thus, it possibly causes the volatilization of some elements (Ares et al. 2012). Oven-drying alters much less the morphological structure of biomonitors, also being eco-friendlier than acid washing (Giordano et al. 2009). A third method, the so-called heat shock treatment (which is carried out at 50–60 °C on wet mosses, lichens and algae) has never been tested in this context (Tretiach et al. 2012; Bertuzzi et al. 2013, 2017).

Another aspect common to these studies is that the accumulation efficiency of living and dead biomonitors is generally tested by transplanting paired living-dead samples at a little number of sites (e.g. Adamo et al. 2007; Giordano et al. 2009; Debén et al. 2016). Therefore, although the experimental design provides with a discrete number of replicates, poor conclusions can be drawn on the potential interpretational bias resulting from the exposure of samples with different health status in a real, large sample-sized survey.

In this work, the hypothesis that living and dead lichen matrices differ in terms of accumulation efficiency is tested using the highly performing lichen bioaccumulator *P. furfuracea*, the only species for which this issue was previously addressed, therefore providing a starting point to perform reliable result comparison. The choice of the species is also dictated by its widespread use in lichen transplants (e.g. Adamo et al. 2003; Cicek et al. 2007; Jozic et al. 2009; Tretiach et al. 2011; Petrova et al. 2015) and its role in methodological studies (e.g. Incerti et al. 2017; Cecconi et al. 2018; Cecconi et al. 2019b, c) that has led to the development of the very last interpretative tool for lichen bioaccumulation data from transplant applications (Cecconi et al. 2019a). Here, for the first time, the issue of *P. furfuracea* vitality in relation to its accumulation capacity is faced in a large-scale transplant application (characterized by a high density of experimental sites), carried out in an area of NE Italy, already used in methodological studies (Kodnik et al. 2015, 2017), adopting a devitalization treatment, which permits to avoid the alteration of the original physical structure and chemical composition of samples, caused by more aggressive procedures (Adamo et al. 2007, 2008). Eventually, this work is also aimed at investigating the potential interpretative bias derived from lichens in different health conditions.

## Materials and methods

### Lichen collection, sample pre-treatment and storage

On December 8, 2016, c. 400 thalli of *Pseudevernia furfuracea* were collected in an acknowledged background area of the Carnic Alps (317614 E, 5148046 N; 1750 m a.s.l.; Cecconi et al. 2018, 2019b).

After the cleaning and selection procedures (for details, see Cecconi et al. 2019b), the bulk material was split into two sets subjected to different storage conditions. A half of thalli was air dried, vacuum-sealed and stored in a freezer at − 20 °C to preserve their vitality (Honegger 2003). The residual material was instead stored in a dark, refrigerated room at c. 10 °C with ambient air humidity higher than 80%, to achieve devitalization (following an in-house developed protocol).

### Storage and post-storage assessment of lichen vitality

During storage and at the end of the storage, the photosynthetic activity of algal populations was occasionally assessed by chlorophyll fluorescence emission (Chl_*a*_F) measurements on terminal lobes of randomly selected thalli, to assess their health status (Tretiach et al. 2007). Chl_*a*_F was assessed in terms of the maximum quantum yield of primary photochemistry in dark adapted samples, using the parameter *F*_v_/*F*_m_ as a proxy for the efficiency of photosystem II (Candotto Carniel et al. 2017).

Dark-stored thalli were air dried at room temperature, whereas thalli stored at − 20 °C were thawed in silica for 24 h. *F*_v_/*F*_m_ values were assessed on 60 lobes per set, each one detached from a randomly chosen thallus, by selecting scarcely isidiate terminal lobes of 2.5-cm length. Prior to the Chl_*a*_F measurements, lobes were hydrated in jars for 48 h at c. 100% relative humidity (RH), 18 °C, and 30 μmol photons m^−2^ s^−1^ for 14 h per day. Once hydrated, lobes were rinsed for 3 min in dH_2_O, gently shaken to remove the excess water, then dark-adapted for 30 min.

Chl_*a*_F measurements were carried out with a Photosynthetic Efficiency Analyzer Fluorimeter Handy-PEA (Hansatech, King’s Lynn, UK). Lichens were considered either fully vital (henceforth, ‘living—*L*—samples’) or dead (henceforth, ‘dead—*D*—samples’) when *F*_v_/*F*_m_ exceeded 0.5 or it was lower than 0.1, respectively (Jensen 2002). As expected, the long-term storage at − 20 °C was effective in preserving the vitality of thalli; contextually, the protracted dark storage at high ambient air humidity led to a successful devitalization (Supplementary Fig. S1).

After the vitality assessment, a sufficient amount of samples from the two bulk sets was selected to assess the elemental composition of living and devitalized lichen material prior the transplant study. These samples were not exposed in the study area (“unexposed” or “pre-exposure” samples), but refrozen at − 20 °C until retrieving transplanted *L* and *D* samples. The study area covers c. 40 km^2^ in a typical mixed land use plain located at the foot of the Carnic Pre-Alps (NE Italy) (Kodnik et al. 2015). It includes a medium-extent urban centre (Maniago) and three smaller centres (Arba, Cavasso Nuovo and Fanna). The main potential anthropogenic pollution sources are a large industrial park, an isolated medium-sized cement plant (Supplementary Fig. S2), vehicular traffic and agricultural activities (Kodnik et al. 2017; Supplementary Methods S1). In the study area, the elemental and PAH deposition patterns were repeatedly assessed through native and transplanted lichens (Tretiach and Baruffo 2001a; Tretiach and Pittao 2008; Kodnik et al. 2015, 2017).

### Study area and lichen transplant

In this study, 40 transplant sites were selected according to the systematic sampling design originally adopted in Kodnik et al. (2015, 2017) (Supplementary Table S1, Supplementary Fig. S2), and located as much as possible far from linear and point emission sources such as busy roads and house chimneys, as possibly acting as confounding agents for the interpretation of results. Thirty-seven sites were located at the knots of a 700-m step grid, and three further in the nearby centres of Arba, Cavasso and Maniago.

A week before the field exposure, *L* and *D* thalli were mounted on exposure devices. From three to six thalli were secured with plastic cable ties to wooden rods (120 cm long, 0.5 cm Ø) previously subjected to dH_2_O washing. Overall, 80 exposure devices were assembled, 40 bearing *L* thalli and 40 bearing *D* ones. Immediately after their preparation (June 13, 2018), paired (*L*-*D*) exposure devices were placed at each transplant site, attached to the external branches of deciduous trees at c. 4 m above the ground, within 8 h of field work. After 8 weeks (August 18th), all samples were retrieved, with the exception of the *D* sample exposed at site 7D that was missing.

After their retrieving, the health status of samples was again assessed by Chl_*a*_F measurement on 60 lobes per set, as described above (Sect. 2.2). After the exposure, *L* samples stayed vital, although *F*_v_/*F*_m_ mean values lowered due to stressing field conditions (Supplementary Fig. S1), in accordance with previous observations on *P. furfuracea* transplanted in summertime, irrespective the pollutant loads (e.g. Tretiach et al. 2007; Pirintsos et al. 2011).

### Sample processing and element content determination

After their retrieving, samples were transported to the laboratory and left to dry out at room temperature for 24 h. Afterwards, terminal lobes homogenous in size (15–25 mm) were selected and grinded for 4 min at 30 Hz with a mixer mill Retsch MM 400. The resulting powder (c. 1 g per sample) was stored in pre-labelled polypropylene tubes and kept in silica until the analytical determination.

Element content determination was performed at Bureau Veritas Mineral (BVM) laboratories (Arkansas, USA). Grinded samples of *P. furfuracea* were subjected to a partial acid digestion with ACS-grade HNO_3_ (1 h), and Aqua Regia (ACS-grade HCl-HNO_3_, volume ratio 1:3) in a boiling water bath (95 °C, 1 h). The concentrations of 24 elements (Al, As, Ba, Bi, Ca, Cd, Co, Cr, Cu, Fe, Hg, K, Mg, Mn, Mo, Na, Ni, P, Pb, S, Sb, Sr, Ti, Zn) were measured through inductively couple plasma mass spectroscopy (ICP-MS), with a PerkinElmer Elan 6000 ICP-MS. The resulting concentration values were expressed on a dry weight basis (μg g^−1^ DW).

In order to assess the accuracy of analytical procedures, BVM laboratories analyzed aliquots of two in-house reference materials (CDV-1 and V16, plant leaves), with the same protocol adopted for the experimental samples. Accuracy results were expressed in terms of mean recovery percentages (Supplementary Table S2).

### Data analysis

Descriptive statistics were calculated for the concentrations of 24 target elements measured in unexposed and exposed *L* and *D* samples (Supplementary Table S3). Afterwards, element concentrations in exposed samples were expressed with respect to those of unexposed ones in terms of the so-called exposed-to-unexposed ratio (*EU* ratio; Cecconi et al. 2019a), and the same descriptive statistics were calculated for dimensionless *EU* ratios.

Explorative multivariate statistics (PCA and hierarchical CA) were performed on the *EU* ratio data matrix of *L* and *D* samples. Firstly, a PCA was performed on the matrix 78 × 24 (39 *L* samples plus 39 *D* samples × 24 elements). The four quantitative levels of the factor ‘land use’ (U, urban; I, industrial; R, rural; N, natural; Supplementary Methods S1.2) were also included in the analysis as supplementary variables and shown as vectors in the principal component (PC) space of elements. The two levels of the factor ‘sample set’ (i.e. *L* and *D*) were inserted as binary dummy variables, indicating the vitality of lichen samples. Dummy and supplementary variables were not used to calculate the principal components (PCs) but plotted on the ordination space based on their correlations with the PCs (Legendre and Legendre 1998).

For comparative purposes, *EU* ratio data derived from living and dead samples were also organized in two distinct matrices 39 × 24 (39 samples × 24 elements for either *L* or *D* sets). The variables (elements) and cases (sites) of such matrices were subjected to hierarchical CAs used as distance measure and clustering algorithm, respectively, Pearson’s 1–*r* and the complete linkage, and the Euclidean distance and the Ward’s method. Then, for the element groups and the site clusters, among-group/among-cluster significant differences were tested by non-parametric Kruskal-Wallis ANOVA and Dunn’s post hoc test.

To address the effect of the lichen vitality on the accumulation of single elements and to assess potential interpretational differences derived by the use of living and dead samples, significant differences between median *EU* ratios of *L* and *D* samples were tested by Wilcoxon signed rank test (the same was done for the median concentrations of unexposed and exposed *L* and *D* sample sets; Supplementary Table S3). The lichen vitality was considered to have a systematic effect when the element-specific *EU* ratio was higher in either *L* or *D* in more than 80% of sites. Accordingly, mean *EU* ratios were used to classify the accumulation of the 24 target elements, either overall or site by site, on the basis of the bioaccumulation scale available for 8-week transplant applications (Supplementary Table S4).

All data analyses and graphics were performed with the software packages QGIS 2.18.17 ‘Las Palmas’, Statistica v. 10 (StatSoft Inc., Tulsa, OK, USA) and R (R Core Team 2013). Statistical significance was tested at *α* = 0.05 in all cases.

## Results

### Multivariate assessments

**Fig. 1:**
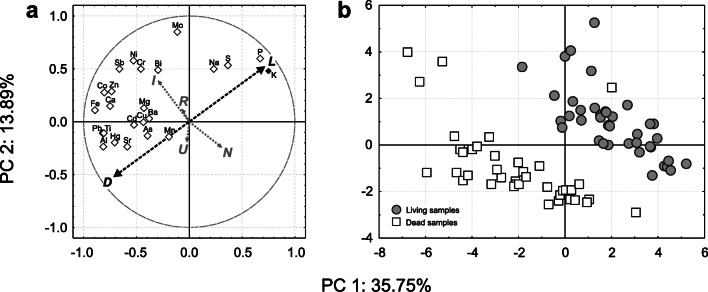
Principal component analysis (PCA) based on *EU* ratio data of elements (a) and *Pseudevernia furfuracea* samples (b). In the principal component space of elements, sample sets and land use categories are represented as supplementary variables (black dotted arrows: *L*—living samples, *D*—dead samples; grey dotted arrows: I—industrial, U—urban, R—rural, N—natural landcover; Supplementary Methods S1.2, Supplementary Table S1)

The first and second principal components (PC 1, PC 2) of the multivariate space describe 35.8% and 13.9% of variance (Fig. 1). PC 1 is negatively associated with the *EU* ratio of most elements (Al, Ca, Cd, Co, Fe, Hg, Pb, Sr, Ti and Zn) and positively with that of K, Na, P and S. Moreover, this axis is negatively and positively correlated with living and dead lichen samples (their projection on PC 1 being ± 0.72), therefore indicating a higher bioaccumulation of elements placed at negative scores of PC 1 in dead samples, and contextual higher *EU* ratios of K, Na, P and S in living samples. PC 2 is instead positively correlated with Bi, Cr, Mo, Ni and Sb (with negative PC 1 scores), as well as with K, Na, S and P (with positive PC 1 scores) (Fig. 1a). Concerning landcover categories in the surroundings of the transplant sites, the industrial land use is respectively negatively/positively correlated with PC 1/PC 2, suggesting an enhanced accumulation (higher *EU* ratios) of Al, Bi, Ca, Cd, Co, Cr, Fe, Hg, Mo, Ni, Pb, Sb, Sr, Ti and Zn. Natural land use is consistently positively/negatively correlated with PC 1/PC 2, suggesting the lowest loss (higher *EU* ratios) of physiological elements (mostly K and P) as well as the lowest accumulation of Bi, Cr, Mo, Ni and Sb.

Lichen samples segregate according to their set, with *L* samples mostly placed in the first quadrant and *D* samples mostly occupying the third quadrant (Fig. 1b). However, there was an exception to this general pattern, i.e. the *D* sample is exposed at site 5A, characterized by an anomalous high S enrichment.

**Fig. 2:**
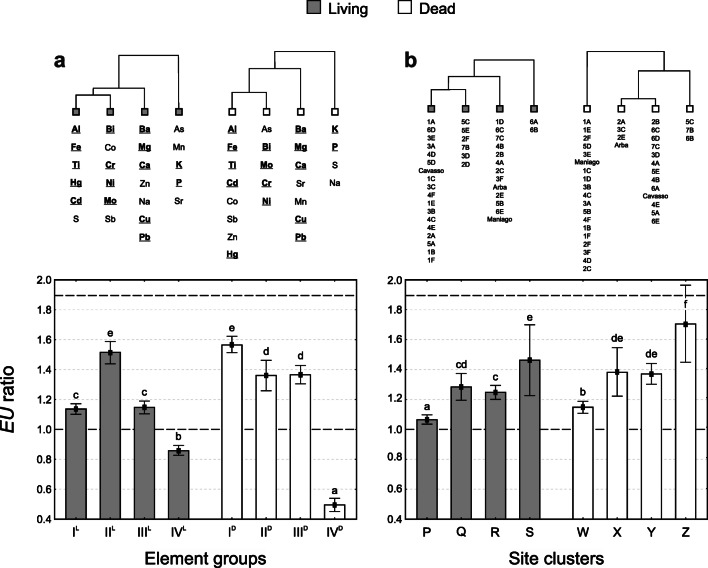
Cluster analysis (CA) of elements (a) and sites (b) with bar charts showing mean *EU* ratio values for different element groups (a) and site clusters (b) (error bars indicate 95% confidence intervals). Black dotted lines show the *EU* thresholds of bioaccumulation classes for 8-week transplants (Supplementary Table S4). Letters above bars indicate among-group/cluster significant differences (Kruskal-Wallis ANOVA and Dunn’s post hoc test). Elements shared by matching groups are underlined and reported in bold

The cluster analysis (CA) of elements performed on the *EU* ratios of *L* and *D* sets produced dendrograms with comparable topologies (Fig. 2a). At the same linkage distance, four groups can be identified in both cases, with elements co-occurring within each group of the two dendrograms. Therefore, matching groups were labelled with the same roman numeral and a superscript reflecting the sample set (I^L^-I^D^, ... IV^L^-IV^D^). In particular, Al, Fe and Ti (lithogenic elements) plus Cd and Hg, Bi, Cr, Mo and Ni (heavy metals associated to steel work industry), Ba, Ca and Mg (alkaline earth metals) plus Cu and Pb, as well as K and P (physiology-related elements), are shared within groups I, II, III and IV, respectively.

The results of the non-parametric Kruskall-Wallis ANOVA reveal that the *EU* ratios of element groups significantly differ among the sample sets (see bar charts at the bottom of Fig. 2a). Lithogenic elements of group I show the largest significant differences between *EU* ratios of *L* and *D* samples, with *D* samples characterized by the highest values, so as group III, although with more limited inter-set differences. Differently, group II shows significantly higher *EU* ratios in *L* samples. Physiological elements of group IV are instead not accumulated (‘Absence of bioaccumulation’) by both sample sets, although their loss is substantially higher in *D* samples (Fig. 2a). Overall, averaged *EU* ratios for different groups of elements in both sample sets never exceed the upper threshold of ‘low bioaccumulation’ class (*EU* ratio ≤ 1.9; class 2 of the bioaccumulation scale; Fig. 2a; Supplementary Table S4), therefore highlighting generally low elemental depositions over the study area.

By cutting the site dendrograms at the same linkage distance, four clusters of comparable dimensions are still formed for either *L* or *D* samples (Fig. 2b). However, in this case, the two dendrograms do not share the same overall topology; therefore, these were labelled with different letters. Although site clusters are comparable in size (P, Q, R and S include 18, 6, 13 and 2 sites respectively, whereas W, X, Y and Z include 19, 4, 13 and 3 sites), a weaker match can be noticed between the cluster composition of *L* and *D* sets (Fig. 2b). In the three clusters sharing sites, mean *EU* ratio values differ among the sample sets with *D* samples exhibiting significantly higher values. Averaged *EU* ratios for different site clusters never exceed the ‘low bioaccumulation’ class (*EU* ratio ≤ 1.9; class 2 of the bioaccumulation scale; Fig. 2b; Supplementary Table S4). In particular, although significantly differing, clusters P and W (sharing the highest number of samples) show the lowest mean *EU* levels. Clusters S and Z (sharing the industrial site 6B) show instead the highest bioaccumulation: in this case, and limited to cluster Z (*D* samples), the upper 95% confidence limit falls in the ‘moderate bioaccumulation’ class (1.9 < *EU* ratio ≤ 2.7; class 3 of the bioaccumulation scale; Fig. 2b; Supplementary Table S4). An intermediate situation can be highlighted for clusters Q and R of *L* samples and for clusters X and Y of *D* samples, respectively. Indeed, within the same sample set, the averaged *EU* ratio values of these clusters do not differ (Fig. 2b).

### Living vs dead: single-element accumulation performance and transplant site alteration

**Fig. 3:**
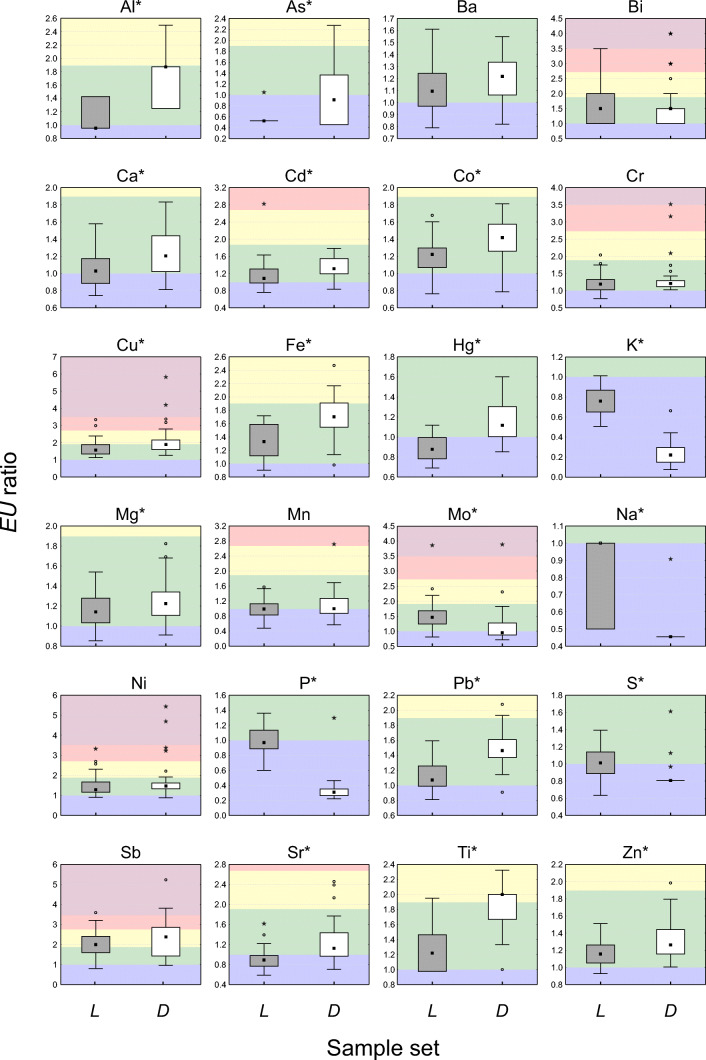
Boxplots of *EU* ratio data of living (*L*, grey) and dead (*D*, white) *Pseudevernia furfuracea* samples for 24 target elements. Data refer to median, first and third quartiles, and non-outlier ranges (outliers and extreme values are highlighted by circles and stars, respectively). Asterisks next to the element name indicate significant differences between the sample sets (Wilcoxon test; Table 1). Background is coloured according to the *EU* range of bioaccumulation classes (Supplementary Table S4)

When addressing single elements, Al, As, Ca, Cd, Co, Cu, Fe, Hg, K, Mg, Mo, Na, P, Pb, S, Sr, Ti, Zn show significant *EU* ratio differences between sample sets (Table 1; Fig. 3).

**Table 1:** Descriptive statistics (mean ± standard deviation, 95% confidence interval, median and range) of element *EU* ratios in living (*L*) and dead (*D*) samples, along with the output of the Wilcoxon test for paired samples on *EU* ratio data (*p* values < 0.05 are reported in italic)

Element	Living samples (*L*)				Dead samples (*D*)				Wilcoxon	
	Mean ± SD	C.I. 95%	Median	Range	Mean ± SD	C.I. 95%	Median	Range	Z	*p* value
Al^+^	1.16 ± 0.24	1.08–1.24	0.95	0.95–1.43	1.73 ± 0.30	1.63–1.83	1.88	1.25–2.50	5.30	*1.1* × *10*^*−7*^
As	0.65 ± 0.22	0.57–0.72	0.53	0.53–1.05	0.99 ± 0.59	0.80–1.18	0.91	0.45–2.27	2.39	*0.017*
Ba	1.11 ± 0.21	1.05–1.18	1.09	0.79–1.61	1.19 ± 0.20	1.12–1.25	1.22	0.82–1.55	1.77	0.076
Bi	1.67 ± 0.77	1.42–1.92	1.50	1.00–3.50	1.58 ± 0.72	1.34–1.81	1.50	1.00–4.00	1.09	0.276
Ca^+^	1.05 ± 0.19	0.99–1.11	1.03	0.74–1.58	1.24 ± 0.28	1.15–1.33	1.20	0.81–1.83	3.88	*1.0 × 10*^*−4*^
Cd	1.18 ± 0.33	1.07–1.29	1.09	0.76–2.83	1.35 ± 0.21	1.28–1.42	1.31	0.83–1.79	3.19	*0.001*
Co	1.20 ± 0.20	1.14–1.27	1.22	0.76–1.68	1.39 ± 0.23	1.32–1.47	1.42	0.79–1.81	3.88	*1.0 × 10*^*−4*^
Cr	1.22 ± 0.27	1.13–1.30	1.19	0.77–2.04	1.35 ± 0.51	1.18–1.51	1.21	1.03–3.53	1.27	0.204
Cu	1.67 ± 0.48	1.52–1.83	1.57	1.12–3.34	2.08 ± 0.85	1.80–2.35	1.89	1.26–5.83	3.06	*0.002*
Fe^+^	1.32 ± 0.25	1.24–1.41	1.33	0.90–1.72	1.71 ± 0.30	1.61–1.81	1.70	0.98–2.47	4.90	*9.7 × 10*^*−7*^
Hg^+^	0.89 ± 0.12	0.85–0.92	0.88	0.69–1.12	1.16 ± 0.20	1.09–1.22	1.11	0.85–1.60	4.94	*7.8 × 10*^*−7*^
K^○^	0.76 ± 0.13	0.72–0.80	0.76	0.51–1.01	0.24 ± 0.11	0.20–0.27	0.22	0.07–0.66	5.44	*5.3 × 10*^*−8*^
Mg	1.16 ± 0.16	1.11–1.22	1.14	0.85–1.54	1.24 ± 0.20	1.18–1.31	1.22	0.91–1.82	2.22	*0.026*
Mn	1.01 ± 0.26	0.91–1.11	0.99	0.48–1.57	1.10 ± 0.37	0.98–1.22	1.00	0.56–2.72	1.16	0.247
Mo^○^	1.54 ± 0.53	1.37–1.71	1.46	0.80–3.87	1.17 ± 0.56	0.99–1.36	0.95	0.71–3.89	4.35	*1.3 × 10*^*−5*^
Na^○^	0.76 ± 0.25	0.67–0.84	1.00	0.50–1.00	0.55 ± 0.19	0.49–0.61	0.45	0.45–0.91	4.48	*7.5 × 10*^*−6*^
Ni	1.46 ± 0.52	1.29–1.63	1.28	0.90–3.33	1.72 ± 0.93	1.42–2.02	1.47	0.88–5.44	1.90	0.058
P^○^	0.98 ± 0.16	0.93–1.04	0.97	0.60–1.36	0.33 ± 0.17	0.28–0.39	0.31	0.22–1.30	5.43	*5.7 × 10*^*−8*^
Pb^+^	1.12 ± 0.17	1.06–1.17	1.07	0.81–1.59	1.48 ± 0.23	1.41–1.56	1.46	0.91–2.08	5.19	*2.1 × 10*^*−7*^
S^○^	1.03 ± 0.18	0.97–1.08	1.01	0.63–1.39	0.85 ± 0.14	0.81–0.90	0.81	0.81–1.61	4.05	*5.2 × 10*^*−5*^
Sb	2.00 ± 0.62	1.80–2.20	2.00	0.80–3.60	2.26 ± 0.86	1.98–2.54	2.38	0.95–5.24	1.91	0.056
Sr	0.91 ± 0.20	0.85–0.97	0.89	0.59–1.62	1.25 ± 0.41	1.11–1.38	1.13	0.70–2.46	4.17	*3.0 × 10*^*−5*^
Ti^+^	1.24 ± 0.25	1.16–1.32	1.22	0.98–1.95	1.91 ± 0.30	1.74–1.99	2.00	1.00–2.33	4.76	*1.9 × 10*^*−6*^
Zn	1.16 ± 0.15	1.12–1.21	1.16	0.93–1.51	1.31 ± 0.20	1.24–1.37	1.26	1.01–1.98	3.52	*4.4 × 10*^*−4*^

^○^Elements showing higher *EU* ratio values in *L* samples in more than 80% of transplant sites (Supplementary Fig. S3)

^+^Elements showing higher *EU* ratio values in *D* samples in more than 80% of transplant sites (Supplementary Fig. S3)

As already highlighted at the cluster level, physiological elements (K, Na, P, S) were generally characterized by ‘absence of bioaccumulation’, but significantly higher elemental losses occurred in *D* samples during the exposure. *L* samples had significant higher *EU* ratios limited to Mo, which, by itself, determined the significant higher median *EU* ratio of group II^L^ (Sect. 3.1; Fig. 2a). Concerning Mg, both sample sets were characterized by ‘low bioaccumulation’, but *D* samples showed slight, although significant, higher *EU* ratios (Fig. 3). Overall, *D* samples were more effective in accumulating lithogenic elements (Al, Ca, Fe, Ti) and As, Cd, Co, Cu, Hg, Pb, Sr and Zn.

A consistent effect of lichen vitality was highlighted over the study area for a subset of elements exhibiting between-set significant differences. Indeed, *EU* ratios of K, Mo, Na, P and S were higher in *L* samples in more than 80% of transplant sites, whereas the opposite was found for Al, Ca, Fe, Hg, Pb and Ti (Supplementary Fig. S3).

When the interpretative scale (Supplementary Table S4) was used to classify the mean *EU* ratios of element content in *L* and *D* samples, this led to different class attribution for some elements (Supplementary Fig. S4). Indeed, the mean *EU* ratio of S in *L* and *D* samples was attributed to ‘low bioaccumulation’ and to ‘absence of bioaccumulation class’, respectively. The opposite was instead observed for Cu, Hg, Sr and Ti. The mean *EU* ratio values calculated for Hg and Sr in *L* samples showed ‘absence of bioaccumulation’ (*EU* ratio ≤ 1; class 1) and ‘low bioaccumulation’ (*EU* ratio ≤ 1.9) in *D* ones, whereas those of Cu and Ti were characterized by ‘low bioaccumulation’ in *L* samples and by ‘moderate bioaccumulation’ (*EU* ratio ≤ 2.7; class 3) for *L* and *D* samples, respectively (Supplementary Fig. S4). With the exception of Cu and Sr, these elements exhibited higher *EU* ratios in more than 80% of transplant sites, in either *L* (for S) or *D* (for Hg and Ti) sample sets.

When *EU* ratios of single elements were addressed site per site, the results obtained by different sample sets depicted a general pattern of low pollutant depositions, irrespective the use of *L* and *D* samples. The majority of transplant sites were accordingly characterized by the predominance of ‘low’ or ‘absence of’ bioaccumulation. Indeed, when considering *L* samples, only two sites out of 39 were characterized by less than 80% of classes 1 and 2 (6A and 4B), whereas 11 sites were exclusively characterized by such classes (Supplementary Fig. S4). When referring to *D* samples, eight sites out of 39 were characterized by less than 80% of classes 1 and 2, whereas six sites were exclusively characterized by such classes (Supplementary Fig. S4).

However, when focusing on the situations of alteration, the use of different sample sets also determines some major differences. Indeed, with *L* samples, only eight sites out of 39 were characterized by more than 10% of classes 3, 4 and 5 (from “moderate” to “extreme” bioaccumulation; Supplementary Fig. S4). Instead, when referring to *D* samples, more than a half of transplant sites were so characterized, with 19 sites showing such pattern (Supplementary Fig. S4).

## Discussion

### Bioaccumulation capacity of living and dead samples

After the exposure in the study area, living and dead *Pseudevernia furfuracea* samples showed different elemental content. Indeed, the statistical analysis of *EU* data characterizing the experimental sets highlighted a higher enrichment of Al, Ca, Fe, Ti, As, Cd, Co, Cu, Hg, Pb, Sr, Zn and a higher loss of K, Na, S and P by dead thalli, whereas living samples were more effective only in accumulating Mo. The latter element is an essential micronutrient for almost all biological systems (especially bacteria, but also eukaryotes), which holds key positions in several enzymes involved in carbon, nitrogen and sulphur metabolism (Peng et al. 2018). However, the role of Mo as enzymatic cofactor, by itself, does not explain an enhanced accumulation by healthy *P. furfuracea* thalli, although suggesting the possibility for interesting in vitro research to clarify the accumulation behaviour of Mo in lichen ecosystems.

The higher loss of K, Na, S and P by dead samples is in accordance with previous observations of impairment of lichen intracellular uptake mechanisms caused by ultrastructural/physiological damage (e.g. Tretiach et al. 2007; Spagnuolo et al. 2011; Corapi et al. 2014). Indeed, when plasma membranes are severely damaged, the cytoplasmatic immobilization of ions by intracellular binding matrix may result impaired (Tyler 1989), causing the loss of ions (Asta and Garrec 1980; Bargagli and Mikhailova 2002).

In lichens, a large proportion of airborne trace elements is mainly accumulated by the extracellular entrapment of particulate matter (Tretiach et al. 2011) occurring within the loose hyphal weft of the medulla, which also prevents toxicity at cell level (Bargagli 1998). Therefore, the relative importance of the particle entrapment in dead matrices may result substantially enhanced due to the empty cell volumes, which also leads to an increased availability of ion binding sites at cell wall level (Richardson et al. 1985). Indeed, lichen cell walls contain a plurality of compounds (e.g. chitin, glucans, polyketides) with several poly-anionic functional groups able to bind metal ions (Sarret et al. 1998). There are several evidences that elements with high affinity for these functional groups, especially Al, Cu, Hg, Fe, Pb and Ti (Nieboer et al. 1978; Bargagli and Mikhailova 2002), may continue to be accumulated in dead thalli (Chettri et al. 1997).

Our findings were in general agreement with the results achieved for other lichen species under different experimental conditions. For instance, Nieboer et al. (1976) investigated the metal uptake by *Umbilicaria muhlenbergii* (Ach.) Tuck. in vitro, proving that the uptake of Ni from solutions of NiCl_2_ was merely physicochemical. Indeed, dead thalli accumulated the metal to a slightly greater extent (Nieboer et al. 1976). In our transplant experiment, after the 8-week exposure, dead *P. furfuracea* samples had higher mean *EU* ratio for Ni, although not significantly (Table 1). Moreover, the metal accumulation was higher in dead samples in 25 out of 39 sites (64% of cases), also producing from single- to three-step differences in bioaccumulation classes at sites 3A, 6B, 4E, 5D, 7B and 5C (i.e. *L* vs *D* samples: classes 2–1, 4–3, 4–2, 3–1, 5–3, respectively).

Chettri et al. (1997) investigated the uptake of Cu, Pb and Zn by *Cladonia convoluta* (Lam.) Anders and *C. rangiformis* Hoffm. from solutions of Pb(NO_3_)_2_, CuCl_2_ and ZnCl_2_. The uptake of Cu and Pb was higher in dead *Cladonia* thalli, whereas the opposite was found for Zn, whose content is usually higher in the intracellular fraction of living thalli (Fortuna et al. 2017). Chettri et al. (1997) also highlighted that Zn suffers competitive uptake, being affected by the co-occurrence of Cu and Pb in the medium. These results match our findings for Cu and Pb; limited to Zn, we highlighted an overall higher accumulation by dead thalli (also revealed by the other single work targeting such issue in *P. furfuracea*; see *infra*). The fully controlled experimental conditions of Chettri et al. (1997), along with the frequently proven species-specificity of elemental accumulation (Nimis et al. 2001; Tretiach and Baruffo 2001b; Minganti et al. 2003; Bergamaschi et al. 2007), may easily explain the discrepancy.

The accumulation efficiency of living and dead *P. furfuracea* was also investigated by Adamo et al. (2007) in a 6-week transplant experiment at two urban sites. The authors demonstrated that the accumulative efficiency of living samples was not higher than that of dead ones, showing the major role of atmospheric particulate, irrespective of organism vitality. For both exposure sites, they reported slightly higher bioaccumulation levels in devitalized samples for Al, Ca, Cd, Cr, Cu, Mn and Zn, with the exception of Hg, instead showing higher levels in living thalli. Overall, our results match previous findings: indeed, all elements showed higher bioaccumulation in dead samples, either significantly (Al, Ca, Cd, Cu and Zn) or not (Cr and Mn, Table 1). The differences observed for Hg may be explained by the interplay of peculiar behaviour of this element in the atmosphere and the different pollutant loads affecting the exposure sites. It is feasible that the low levels observed in this work and by Adamo et al. (2007) may derive from different relative contributions of Hg forms (i.e. gaseous or associated to particulate; Bargagli 2016; Keeler et al. 1995). If so, gaseous Hg would be mostly actively accumulated at intracellular level (Rinino et al. 2005), resulting in an enhanced bioaccumulation by living thalli (as in Adamo et al. 2007), whereas the accumulation of Hg adsorbed on airborne particulate matter would be enhanced in dead matrices by physical entrapment (as possibly in this study).

### Pollutant loads and lichen health: the risk of interpretative bias

Overall, the study area was not exposed to high pollutant loads. Indeed, when classified according to the new interpretative scale for lichen transplants, the majority of *EU* ratio values of both living and dead samples were associated to ‘low’ or ‘absence of’ bioaccumulation classes (*EU* ratio ≤ 1.9). Only Cu and Ti in dead samples, as well as Sb in both living and dead ones exceeded class 2 (Supplementary Table S4 and Fig. S3). Therefore, besides the cautious terminology of the interpretative scale (focusing on lichens—i.e. ‘bioaccumulation levels’—rather than on ‘environmental alteration’; Cecconi et al. 2019a), the historically acknowledged link between lichen elemental enrichment and air pollution (e.g. Herzig et al. 1989; Sloof 1995; van Dobben et al. 2001) expressly indicates the absence of any clear emission pattern in the study area. This is especially true for As, Hg and Pb, elements whose atmospheric concentration is targeted by the European Air Quality Directives (2008/50/EC4, 2004/107/EC5). Despite such general pattern, a small set of elements—Bi, Cu, Ni, and Sb—was characterized by substantial bioaccumulation (*EU* ratio > 2.7; class 3) at several sites in either living or dead lichens. Limited to Sb, it must be acknowledged that its recovery is far from being satisfactory (47.6%; Supplementary Table S2), indicating a substantial underestimation of lichen enrichment (and thus of Sb pollution) in the study area. Instead, Cd, Cr and Ti showed such levels limited to single sites in living samples (Cd: class 4 at Maniago; Cr: class 3 at 6A; Ti: class 3 at 2D), or to a higher number of sites in dead samples (Cr: class 3 at 4E, class 4 at 5C and class 5 at 7B; Ti class 3 at 20 sites; Supplementary Fig. S4). All such elements are generally considered tracers of coal combustion (Van de Velde et al. 1999), also related to iron, steel and ferro-alloy industrial processing (Tretiach and Pittao 2008; Brunialti and Frati 2014). Consistently, Cr, Mo, Ni and Sb showed the highest bioaccumulation levels within or near the industrial park, along with the highest concentrations of Fe, Pb and Zn, however characterized by negligible depositions over the whole territory.

Despite an overall accordance of results obtained by using different sample sets, interpretative differences arise in terms of depositional patterns (which is substantiated by different structures of the two site dendrograms; Fig. 2b) and severity of metal-rich particulate pollution (signals are higher in devitalized samples). In this respect, and concerning Fe, Hg and Pb, it is worth to notice that the pre-exposure concentration values of these elements significantly differ between the experimental sample sets (Supplementary Table S3). In particular, the concentration values of Fe and Hg in unexposed *L* samples (U_*L*_) were slightly, but significantly higher than those of *D* samples (U_*L*_ > U_*D*_), whereas the concentrations measured in exposed (E) *L* and *D* samples were fully comparable (E_*L*_ ≈ E_*D*_). Likely, the different Fe and Hg content between U_*L*_ and U_*D*_ samples should be traced back to the inherent biological variability of the bulk material from which living and dead samples were derived. In the case of Pb, besides higher pre-exposure concentration values in *L* samples, also the concentrations of exposed samples differ, exhibiting the opposite pattern (U_*L*_ > U_*D*_ and E_*L*_ < E_*D*_). Strictly speaking, in case of limited elemental depositions, as in this case, significant differences observed in *EU* ratio denominators (*U* values) would not allow proper ratio comparison. Indeed, significantly higher denominators in *L* samples could, by themselves, produce lower *EU* ratios for such samples, possibly determining unreliable outcomes of statistical testing for such elements. Nonetheless, it must also be considered that, in such cases, *EU* ratios were higher in *D* samples in more than 80% of transplant sites (Table 1; Supplementary Fig. S3), and that *EU* ratios of *L* and *D* samples determined different bioaccumulation classes for 33%, 64% and 28% of sites (Supplementary Fig. S4). Since we confidently exclude that any differential contamination of sample sets may have occurred in the laboratory, this has to be regarded as an indication that the effect of the pre-exposure physiological status of lichen thalli likely overcomes that of different pre-exposure concentrations.

Another aspect that has to be taken into account is that alive thalli exposed in the study area may experience different degrees of physiological impairment (see the variation of *F*_v_/*F*_m_ distributions in living samples before and after exposure; Supplementary Fig. S1), depending on the site-specific environmental conditions and the initial health status of each thallus (Piccotto and Tretiach 2010; Piccotto et al. 2011). It is possible to assume that when the initial physiological differences among transplanted samples are stronger, also the ‘noise’ associated to their ‘bioaccumulation signal’ is higher. Indeed, our data suggest that samples in different physiological conditions produced different results in terms of bioaccumulation, and this may introduce an additional, undesirable source of variability affecting the following interpretation of the results. Unfortunately, transplant biomonitoring over large areas is associated to the regular testing of lichen vitality only exceptionally (e.g. Corapi et al. 2014). Hence, devitalization of sample before exposure might be the best solution to decrease the variability of the results ascribable to possible variations in sample vitality during the exposure, maximizing the ‘signal-to-noise’ ratio.

## Conclusions

This work is the first attempt to test the bioaccumulation performance of living vs dead samples of *Pseudevernia furfuracea*, in a side-by-side transplanting at 40 sites in a large, mixed land use area of NE Italy. Overall, dead thalli accumulated higher amounts of elements of environmental concern (e.g. As, Cd, Hg and Pb) as well as those of soil origin (e.g. Al, Ca, Fe and Ti), thus providing a further indication that in transplanted lichens, passive uptake mechanisms play a major role in accumulation of trace elements. Non-negligible interpretational discrepancies in terms of different bioaccumulation classes for 80% of the exposure sites were observed, suggesting that the progressive physiological impairment of living thalli caused by a prolonged exposure to unfavourable environmental conditions might be an important, undesirable source of noise that should preferably be avoided. The use of devitalized samples could reduce this noise, decreasing in the meantime sampling and storage cost. However, the proposal of introducing devitalization as a routine procedure in protocols of lichen transplants (as done for mosses) needs to be sustained by further investigation on the (bio-)degradation resistance of dead thalli, although in this respect our results are fully supportive.

## Supplementary information

ESM 1 (PDF 994 kb)

## Data Availability

The datasets generated and/or analyzed during the current study are property of Buzzi Unicem S.p.A. (Casale Monferrato, Italy); they are available from the corresponding author who will inform Buzzi Unicem that the data will be released on reasonable request.
