# Supplementary material for: Element accumulation performance of living and dead lichens in a large-scale transplant application
Source: Environ Sci Pollut Res Int. 2020 Dec 4;28(13):16214–26. doi: 10.1007/s11356-020-11797-7 (PMC7969570; doi:10.1007/s11356-020-11797-7)
Supplement: Supplementary file 1 — (PDF 994 kb) [file 11356_2020_11797_MOESM1_ESM.pdf]

Element accumulation performance of living and dead lichens in a large-scale transplant application

Elva Cecconi[1§]; Lorenzo Fortuna[2§]; Marco Peplis[1]; Mauro Tretiach[1*]

[1]Department of Life Sciences, University of Trieste, Via L. Giorgieri 10, 34127 Trieste, Italy

[2]Department of Chemical and Pharmaceutical Science, University of Trieste, Via L. Girogieri 1, 34127 Trieste, Italy

§ These authors contributed equally to this work

* Corresponding author:

Prof. Mauro Tretiach,

tretiach@units.it

Via L. Giorgieri 10,

34127 Trieste, Italy

SUPPLEMENTARY MATERIAL

SUPPLEMENTARY METHODS S1.1-S1.2

SUPPLEMENTARY TABLES S1-S4

SUPPLEMENTARY FIGURES S1-S4

SUPPLEMENTARY REFERENCES

Supplementary Methods S1

S1.1 The study area

The study area covers c. 43.6 km^2, and it is located at the foot of the Carnic Pre-Alps, including the municipalities of Fanna, Maniago, Cavasso Nuovo and Arba (Supplementary Fig. S2). According to the Köppen-Geiger climate classification, the study area has a temperate oceanic climate, with mean annual temperature and precipitation respectively ranging between 11.5 and 13.5 °C, and 1400 to 2000 mm (with c. 110 rainy days per year; Kottek et al., 2006; ARPA FVG, 2015). From a pedological point of view, the majority of the study area is characterized by silty-clay subalkaline soils, generally very drained. Limited to the central-northern portion of the study area, soils are mostly alluvial. The land use of the study area is quite heterogeneous, being characterized by a patchwork of forested, agricultural, urban and industrial areas (Bossard et al., 2000; Supplementary Table S2). The southern and eastern parts of the study area are mostly characterized by agricultural land use (maize, forage, and rapeseed crops) with the occurrence of several zootechnical activities. In the northern part, the urban land use prevails; the largest town is the municipality of Maniago (c. 11,700 inhabitants), connected to neighbouring smaller municipalities by a capillary road system (Kodnik et al., 2015, 2017).

The main point pollution source is a cement plant located in the municipality of Fanna (Buzzi Unicem SpA, 2345474 E, 5116064 N; Supplementary Fig. S2), and a large industrial park of 1.6 km^2 located in the south-western part of the study area (Supplementary Fig. S2), which hosts steel, metallurgical, and metal-alloy transformation activities for the production of aeronautical, medical and civil manufactures.

S1.2 Land use characterization of the transplant sites

The 40 transplant sites were characterized in terms of land use using the Corine Land Cover map 2012 (Bossard et al., 2000) in GIS environment. The percent cover of urban (2nd order level ‘11’), industrial (2nd order level ‘12’), rural (1st order level ‘2’) and natural (2nd order level ‘31’) landcover was estimated within buffers of 125 m radius centred on each site. Sites were accordingly classified as urban, industrial, rural or natural when the percent cover of the corresponding class was higher than 50% (Supplementary Table S1).

Supplementary Tables S1-S4

Supplementary Table S1. List of transplant sites with alphanumeric identification codes (site ID), UTM coordinates, altitude and land use classification obtained considering the percent coverage of different CLC categories in circular buffers of 125 m radius centred in the transplant sites (Supplementary methods S1.1).

Site ID	UTM coordinates		Altitude (m a.s.l.)	Land use
	E	N		
1A	2343347	5116507	279	Natural
1B	2344025	5116735	324	Natural
1C	2344633	5116969	382	Natural
1D	2345383	5116934	299	Natural
1E	2346008	5117171	279	Urban
1F	2346755	5117276	270	Urban
2A	2343465	5115852	276	Urban
2B	2344060	5116095	270	Rural
2C	2344853	5116292	264	Rural
2D	2345549	5116006	253	Rural
2E	2346153	5116443	257	Rural
2F	2346845	5116601	254	Rural
3A	2343651	5115202	276	Urban
3B	2344315	5115216	267	Rural
3C	2344981	5115604	259	Rural
3D	2345739	5115575	248	Rural
3E	2346314	5115862	241	Rural
3F	2347092	5115850	242	Rural
4A	2343794	5114313	275	Urban
4B	2344503	5114672	263	Rural
4C	2345117	5114753	253	Rural
4D	2345854	5114939	236	Rural
4E	2346543	5115093	231	Rural
4F	2347088	5115190	229	Rural
5A	2343667	5113708	275	Rural
5B	2344595	5114199	264	Rural
5C	2345243	5114040	253	Rural
5D	2346052	5114223	240	Rural
5E	2346677	5114436	228	Rural
6A	2343855	5112999	268	Industrial
6B	2344703	5113241	257	Industrial
6C	2345404	5113217	248	Rural
6D	2346009	5113620	241	Rural
6E	2346775	5113701	231	Rural
7B	2344650	5112723	258	Rural
7C	2345533	5112752	245	Urban
7D	2346373	5112839	232	Rural
Arba	2349472	5112557	210	Urban
Cavasso Nuovo	2348230	5118263	278	Urban
Maniago	2341823	5114748	303	Urban

Supplementary Table S2. Lower Limit of Detection (LoD; μg g^{-1}) and recovery percentages (calculated as the percentage ratio between the measured and the expected values) for two in-house standard materials (i.e., V16 and CDV-1). Recovery percentages lower than 70% are highlighted in bold.

Element	LoD	Standard materials	
		V16	CDV-1
Al	100	95.4	86.7
As	0.1	89.6	82.1
Ba	0.1	92.4	98.2
Bi	0.02	-	-
Ca	100	96.7	93.3
Cd	0.01	89.2	83.3
Co	0.01	76.6	86.3
Cr	0.1	72.6	91.5
Cu	0.01	80.1	84.5
Fe	10	81.0	95.8
Hg	0.001	100. 7	108.9
K	100	90.9	87.0
Mg	10	94.0	96.4
Mn	1	90.7	94.5
Mo	0.01	75.4	85.0
Na	10	**66. 7**	96.2
Ni	0.1	77.1	83.3
P	10	97.1	73.6
Pb	0.01	93.1	86.0
S	500	-	83.3
Sb	0.02	**47.6**	-
Sr	0.5	95.8	96.5
Ti	1	82.6	74.4
Zn	0.1	85.3	86.3

Supplementary Table S3. Descriptive statistics (mean ± standard deviation, 95% confidence interval, median and range) of element concentration data (µg g^{-1}) in living and dead *Pseudevernia furfuracea* samples, along with the output of the Wilcoxon test for paired samples (*p*-values < 0.05 are reported in italic). Element content values for unexposed (n = 10) and exposed samples (n = 39) are reported in the first and second row, respectively.

Element	Living samples				Devitalized samples				Wilcoxon	
	**Mean ± SD**	**C.I. 95%**	**Median**	**Range**	**Mean ± SD**	**C.I. 95%**	**Median**	**Range**	**Z**	***p*-value**
Al	210 ± 32	187 – 233	200	200 - 300	160 ± 52	123 - 197	200	100 - 200	1.83	0.068
	244 ± 50	227 - 260	200	200 - 300	277 ± 48	261 - 293	300	200 - 400	2.49	*0.013*
As	0.19 ± 0.07	0.14 - 0.24	0.20	0.10 - 0.3	0.22 ± 0.16	0.10 - 0.34	0.15	0.10 - 0.60	0.07	0.944
	0.12 ± 0.04	0.11 - 0.14	0.10	0.10 - 0.20	0.22 ± 0.13	0.18 - 0.26	0.20	0.10 - 0.50	3.39	*0.001*
Ba	12.8 ± 2.2	11.2 - 14.3	12.8	9.9 - 16.4	13.6 ± 2.7	11.7 - 15.4	12.9	10.3 - 19.4	0.87	0.386
	14.3 ± 2.7	13.4 - 15.1	14.0	10.1 - 20.6	16.1 ± 2.7	15.2 - 16.9	16.5	11.1 – 21.0	3.21	*0.001*
Bi	0.02 ± 0.00	-	0.02	0.02 - 0.02	0.02 ± 0.00	-	0.02	0.02 - 0.02	0.00	-
	0.03 ± 0.02	-	0.03	0.02 - 0.07	0.03 ± 0.01	-	0.03	0.02 - 0.08	1.17	0.242
Ca	4180 ± 922	3520 - 4840	4300	2700 - 5500	3820 ± 935	3151 - 4489	3500	2600 - 5800	0.82	0.415
	4377 ± 799	4118 - 4636	4300	3100 - 6600	4738 ± 1078	4389 - 5088	4600	3100 - 7000	2.14	*0.032*
Cd	0.09 ± 0.01	0.08 - 0.10	0.09	0.07 - 0.11	0.08 ± 0.01	0.08 - 0.09	0.09	0.06 - 0.10	1.48	0.138
	0.11 ± 0.03	0.10 - 0.12	0.10	0.07 - 0.26	0.11 ± 0.02	0.11 - 0.12	0.11	0.07 - 0.15	1.47	0.142
Co	0.13 ± 0.03	0.11 - 0.15	0.14	0.08 - 0.16	0.13 ± 0.02	0.11 - 0.14	0.13	0.09 - 0.16	0.36	0.721
	0.16 ± 0.03	0.15 - 0.17	0.16	0.10 - 0.22	0.18 ± 0.03	0.17 - 0.19	0.18	0.10 - 0.23	3.31	*0.001*
Cr	2.4 ± 0.3	2.1 - 2.6	2.3	2.0- 3.0	2.2 ± 0.3	2.0 - 2.4	2.2	1.9 - 2.7	0.51	0.612
	2.9 ± 0.6	2.7 - 3.1	2.8	1.8 - 4.8	3.0 ± 1.1	2.6 - 3.4	2.7	2.3 - 7.9	0.09	0.925
Cu	4.10 ± 0.98	3.40 - 4.80	3.86	3.21 - 6.39	3.51 ± 0.49	3.16 - 3.86	3.37	2.98 - 4.52	1.27	0.203
	6.87 ± 1.95	6.23 - 7.50	6.43	4.61 - 13.72	7.28 ± 3	6.31 - 8.25	6.64	4.41 - 20.45	0.54	0.591
Fe	233 ± 33	209 – 257	235	190 - 290	194 ± 32	171 - 217	195	150 - 250	2.07	*0.038*
	309 ± 58	290 – 327	310	210 - 400	332 ± 59	313 - 351	330	190 - 480	1.92	0.055
Hg	0.155 ± 0.018	0.142 - 0.168	0.150	0.136 - 0.200	0.118 ± 0.014	0.107 - 0.128	0.122	0.097 - 0.136	2.80	*0.005*
	0.137 ± 0.019	0.131 - 0.143	0.136	0.107 - 0.173	0.136 ± 0.023	0.128 - 0.143	0.131	0.100 - 0.188	0.59	0.558
K	2770 ± 221	2612 - 2928	2750	2500 - 3300	2720 ± 103	2646 - 2794	2700	2600 - 2900	0.41	0.683
	2100 ± 355	1985 - 2215	2100	1400 - 2800	646 ± 309	546 - 746	600	200 - 1800	5.44	*5.3×10-8*
Mg	727 ± 65	681 – 773	735	580 - 830	768 ± 121	681 - 855	785	600 - 930	0.97	0.333
	846 ± 120	807 - 885	830	620 - 1120	956 ± 156	905 - 1006	940	700 - 1400	3.79	*1.5×10-4*
Mn	94 ± 21	79 – 109	99	48 - 128	108 ± 34	84 - 132	116	65 - 173	1.07	0.285
	93 ± 24	86 - 101	93	45 - 148	119 ± 40	106 - 132	108	61 - 294	2.91	*0.004*
Mo	0.14 ± 0.02	0.12 - 0.15	0.14	0.11 - 0.17	0.13 ± 0.02	0.11 - 0.14	0.13	0.10 - 0.15	1.36	0.173
	0.21 ± 0.07	0.19 - 0.23	0.20	0.11 - 0.53	0.15 ± 0.07	0.13 - 0.17	0.12	0.09 - 0.49	4.76	*1.9×10-6*
Na	20 ± 0	-	20	20 - 20	22 ± 4	19 - 25	20	20 - 30	1.34	0.180
	15 ± 5	-	20	10 - 20	12 ± 4	11 - 13	10	10 - 20	2.48	*0.013*
Ni	0.8 ± 0.2	0.7 - 0.9	0.7	0.6 - 1.2	0.7 ± 0.1	0.6 - 0.8	0.7	0.6 - 0.9	1.40	0.161
	1.1 ± 0.4	1.0 - 1.3	1.0	0.7 - 2.6	1.2 ± 0.6	1.0 - 1.4	1.0	0.6 - 3.7	0.81	0.416
P	485 ± 115	402 – 568	475	370 - 770	454 ± 75	401 - 507	455	340 - 600	0.65	0.515
	477 ± 77	452 - 502	470	290 - 660	151 ± 76	126 - 176	140	100 - 590	5.43	*5.7×10-8*
Pb	1.84 ± 0.23	1.67 – 2.00	1.82	1.47 - 2.18	1.50 ± 0.17	1.38 - 1.62	1.49	1.30 - 1.86	2.19	*0.028*
	2.05 ± 0.32	1.95 - 2.16	1.97	1.49 - 2.93	2.22 ± 0.34	2.11 - 2.33	2.19	1.36 - 3.11	2.34	*0.019*
S	790 ± 208	641 - 939	800	500 - 1200	620 ± 123	532 - 708	600	500 - 800	1.58	0.114
	810 ± 139	765 - 855	800	500 - 1100	528 ± 89	499 - 557	500	500 - 1000	4.74	*2.1×10-6*
Sb	0.03 ± 0.01	0.02 - 0.03	0.02	0.02 - 0.04	0.02 ± 0.00	0.02 - 0.02	0.02	0.02 - 0.03	1.60	0.109
	0.05 ± 0.02	0.04 - 0.06	0.05	0.02 - 0.09	0.05 ± 0.02	0.04 - 0.05	0.05	0.02 - 0.11	1.10	0.271
Sr	14.6 ± 4.4	11.5 - 17.8	13.7	10.1 - 22.5	11.8 ± 2.7	9.8 - 13.8	11.5	7.9 - 17.4	1.48	0.139
	13.3 ± 2.9	12.4 - 14.2	13.0	8.6 - 23.7	14.7 ± 4.8	13.1 - 16.3	13.3	8.3 - 29	1.55	0.121
Ti	4.1 ± 0.7	4.0 – 5.0	4.0	3.0 – 5.0	3.3 ± 0.5	3.0 - 4.0	3.0	3.0 - 4.0	1.94	0.052
	5.1 ± 1.0	5.0 - 5.0	5.0	4.0 - 8.0	6.3 ± 0.9	5.0 - 6.0	6.0	3.0 - 7.0	1.73	0.083
Zn	20.9 ± 2.2	19.4 - 22.5	21.4	16.8 - 24.2	19.1 ± 1.9	17.8 - 20.4	19.3	15.7 - 21.6	1.38	0.169
	24.4 ± 3.2	23.3 - 25.4	24.2	19.4 - 31.6	25 ± 3.9	23.7 - 26.3	24.1	19.2 - 37.9	0.68	0.494

Supplementary Table S4. *EU* ratio, percentile-based, five-class interpretative scale for bioaccumulation data (i.e., Bioaccumulation Scale) for lichen transplants (Cecconi et al., 2019). Class codes, description and abbreviations, percentile thresholds, and the corresponding *EU* values associated with bioaccumulation classes are reported.

**Bioaccumulation class**			**Percentile thresholds**	***EU* ratio (8 weeks)**
**ID**	**Description (abbreviation)**			
1	Absence of bioaccumulation	(A)	≤ 25th	≤ 1.0
2	Low bioaccumulation	(L)	(25th, 75th]	(1.0, 1.9]
3	Moderate bioaccumulation	(M)	(75th, 90th]	(1.9, 2.7]
4	High bioaccumulation	(H)	(90th, 95th]	(2.7, 3.5]
5	Severe bioaccumulation	(S)	> 95th	> 3.5

SUPPLEMENTARY FIGURES S1-S4

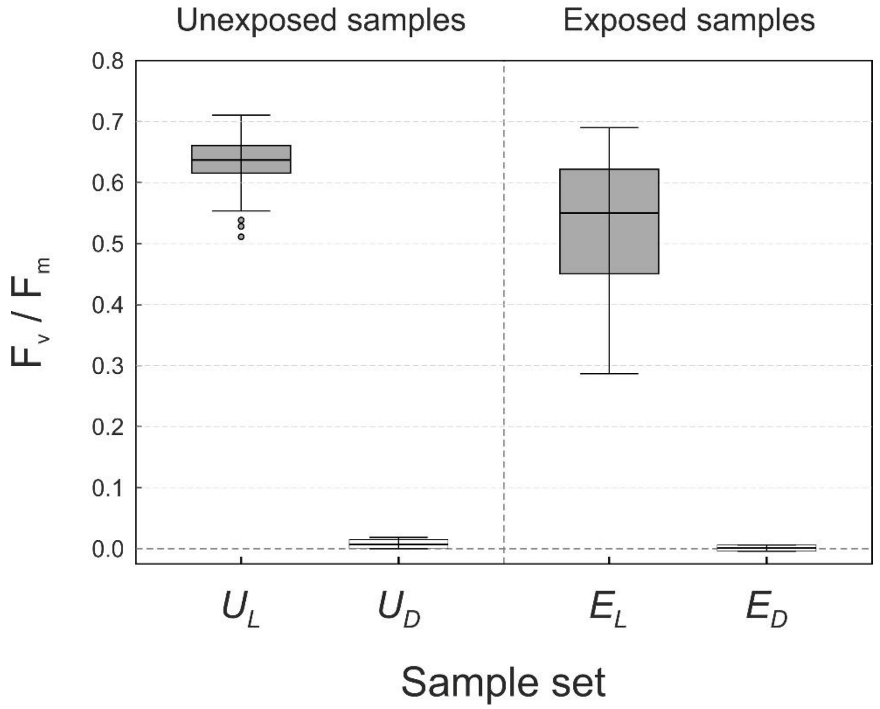

Supplementary Figure S1. Results of the post-storage Chl$_a$F assessment on unexposed and exposed sample sets (U_L: unexposed living samples, i.e. stored for 18 months at -20°C; U_D: unexposed devitalized samples, i.e. dark-stored at c. 10°C for 18 months; E_L: living samples exposed for 6 weeks in the study area; E_D: devitalized samples exposed for 6 weeks in the study area). Boxplots show median F_v/F_m values, interquartile ranges, minima and maxima, and outlier values (circles).

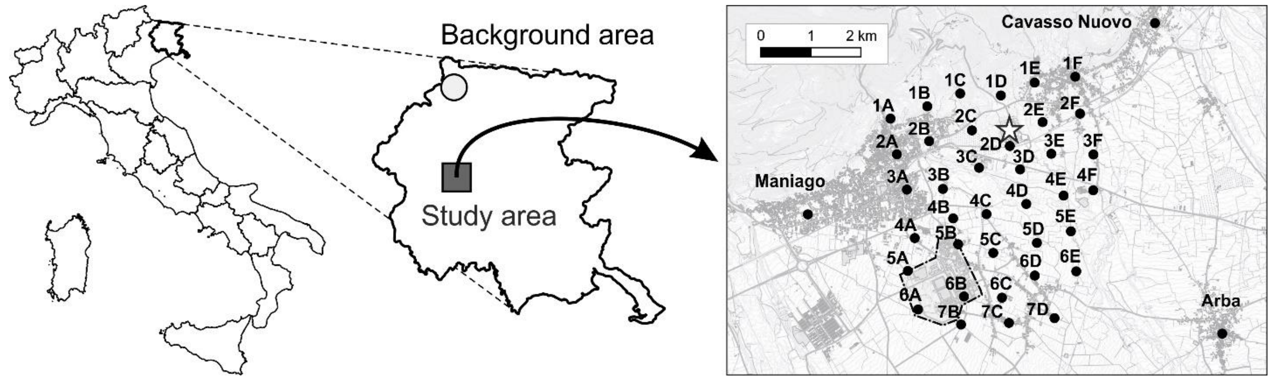

Supplementary Figure S2. Localization of the study area and the background area (for the collection of lichen samples), with indication of the transplant sites of *Pseudevernia furfuracea* labelled by alphanumeric codes (1A - 7D plus Arba, Cavasso Nuovo and Maniago). The two main putative pollution sources, a medium-sized cement plant and an industrial park, are respectively indicated by a star and a closed dashed line.

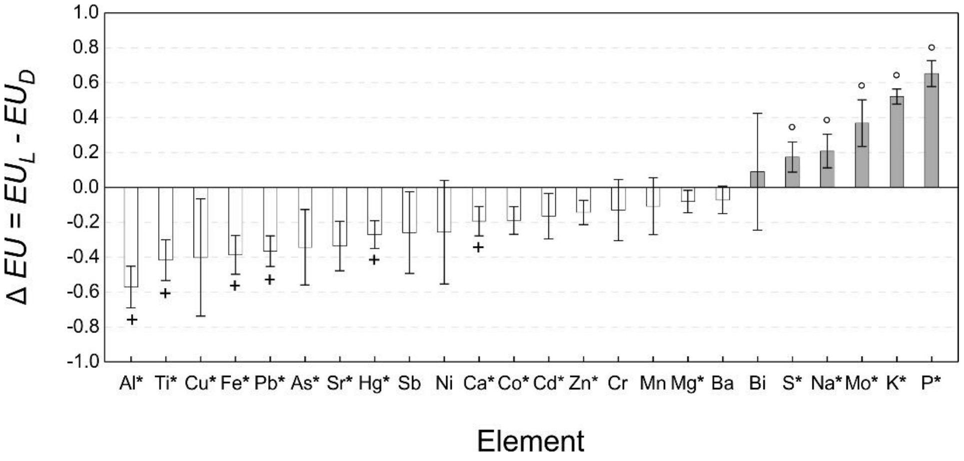

Supplementary Figure S3. Bar charts showing mean differences between *EU* ratio values of living (*L*) and dead (*D*) *P. furfuracea* samples (Δ *EU*) (error bars indicate 95% confidence intervals). Elements showing higher *EU* ratio values in more than 80% of transplant sites in either *L* or *D* samples are respectively marked with circles or crosses. Asterisks next to element labels indicate significant differences between the sample sets, according to the Wilcoxon signed rank test for paired samples (*cf.* Fig. 3 and Table 1).

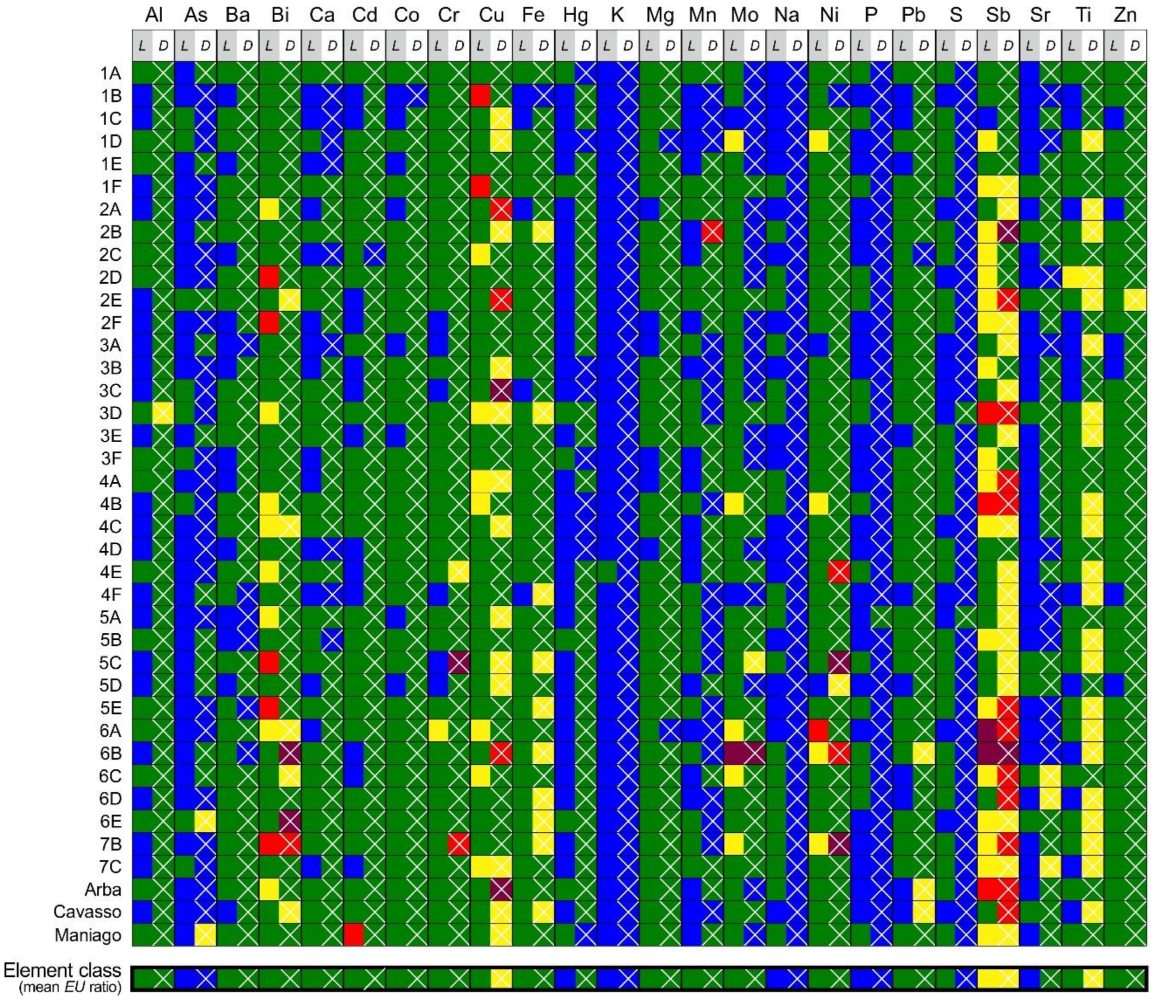

Overall %

1+2		3+4+5	
L	D	L	D
100	100	0	0
95.8	100	4.2	0
100	95.8	0	4.2
87.5	91.7	12.5	8.3
100	100	0	0
91.6	95.9	8.4	4.1
95.8	87.5	4.2	12.5
95.8	79.1	4.2	20.9
91.7	100	8.3	0
87.5	95.8	12.5	4.2
95.8	79.1	4.2	20.9
91.6	95.9	8.4	4.1
100	91.6	0	8.4
95.8	95.8	4.2	4.2
100	91.7	0	8.3
87.5	79.2	12.5	20.8
100	91.6	0	8.4
95.8	100	4.2	0
91.7	91.7	8.3	8.3
79.1	91.7	20.9	8.3
91.7	83.3	8.3	16.7
100	100	0	0
95.8	83.3	4.2	16.7
100	87.5	0	12.5
95.9	91.7	4.1	8.3
95.8	91.7	4.2	8.3
95.8	70.8	4.2	29.2
100	87.5	0	12.5
91.6	87.5	8.4	12.5
75	87.5	25	12.5
87.5	66.7	12.5	33.3
87.5	87.5	12.5	12.5
100	83.4	0	16.6
95.8	79.1	4.2	20.9
83.4	75	16.6	25
91.7	83.3	8.3	16.7
91.7	91.7	8.3	8.3
100	75	0	25
91.6	87.5	8.4	12.5

Supplementary Figure S4. Classification of the transplant sites according to the analytical results in living (*L*, unmarked cells) and devitalized (*D*, cross-marked cells) samples, as resulting from the Bioaccumulation Scale for 8-week transplants (Supplementary Table S4). The element class, calculated by averaging *EU* ratios by column, is reported at the bottom of the figure (element class). The overall percent of elements in classes 1 and 2, as well as in classes 3, 4 and 5, is also reported site per site (limited to the sum of classes 3-5, percentages higher than 10% are highlighted in red).

SUPPLEMENTARY REFERENCES

ARPA FVG (2015) http://www.meteo.fvg.it/clima/clima_fvg/02_documenti_descrittivi_e_approfondimenti/01_Il_clima_del_Friuli_Venezia_Giulia/clima_fvg-divulgativo.pdf

Bossard M, Feranec J, Otahel J (2000) CORINE Land Cover Technical Guide - Addendum 2000. Technical Report 40, European Environment Agency, Copenhagen.

Cecconi E, Fortuna L, Benesperi R, Bianchi E, Brunialti G, Contardo T, Di Nuzzo L, Frati L, Monaci F, Munzi S, Nascimbene J, Paoli L, Ravera S, Vannini A, Giordani P, Loppi S, Tretiach M (2019) New interpretative scales for lichen bioaccumulation data: the Italian proposal. *Atmosphere* 10, 136-154.

Kodnik D, Candotto Carniel C, Licen S, Tolloi A, Barbieri P, Tretiach M (2015) Seasonal variations of PAHs content and distribution patterns in a mixed land use area: A case study in NE Italy with the transplanted lichen Pseudevernia furfuracea. *Atmos Environ* 113, 255-263.

Kodnik D, Winkler A, Candotto Carniel F, Tretiach M (2017) Biomagnetic monitoring and element content of lichen transplants in a mixed land use area of NE Italy. *Sci Total Environ* 595, 858-867.

Kottek M, Grieser J, Beck C, Rudolf B, Rubel F (2006) World map of the Köppen-Geiger climate classification updated. *Meteorologische Zeitschrift* 15, 259-263.
